# Case Report: Pharmacological decision-making in pregnant women with neuromyelitis optica spectrum disorder: two contrasting clinical scenarios

**DOI:** 10.3389/fphar.2026.1885602

**Published:** 2026-07-28

**Authors:** Chun-Fei Wang, Yu-Fei Zhang, Qiang Wei, Yun-Hui Gong

**Affiliations:** 1 Department of Obstetrics and Gynecology, West China Second University Hospital, Sichuan University, Chengdu, China; 2 Key Laboratory of Birth Defects and Related Diseases of Women and Children, Sichuan University, Ministry of Education, Chengdu, China

**Keywords:** AQP4-IgG, azathioprine, methylprednisolone, MOG-IgA, neuromyelitis optica spectrum disorder, pharmacological management, prednisolone, pregnancy

## Abstract

**Background:**

Neuromyelitis optica spectrum disorder (NMOSD) is a relapsing autoimmune disease of the central nervous system that predominantly affects women of reproductive age. Pregnancy requires careful pharmacological decision-making because maternal relapse can lead to severe neurological disability, while fetal exposure to immunosuppressive therapy remains an important safety concern.

**Case presentation:**

We report two contrasting clinical scenarios in pregnant women with NMOSD. Case 1 involved a 30-year-old primigravida with AQP4-IgG-negative NMOSD and weak isolated MOG-IgA positivity of uncertain clinical significance. At 11 weeks of gestation, she developed a disabling relapse with severe bilateral lower-limb weakness, sensory disturbance, inability to walk independently, and urinary dysfunction. High-dose intravenous methylprednisolone led to partial recovery. After multidisciplinary counseling, she chose pregnancy termination at 18+3 weeks because of residual disability, concerns about fetal drug exposure, possible need for further immunosuppression, and anticipated rehabilitation burden. Case 2 involved a 35-year-old primigravida with AQP4-IgG-positive NMOSD who had been clinically stable for approximately 1 year before conception while receiving prednisolone 10 mg/day and azathioprine 50 mg/day. This regimen was continued during pregnancy with neurological, obstetric, and clinical pharmacology monitoring. She remained relapse-free and delivered a healthy female infant at 38 weeks. The infant weighed 3,100 g, had Apgar scores of 10, 10, and 10 at 1, 5, and 10 min, and showed normal growth and development at 12 months.

**Conclusion:**

These cases are not directly comparable and cannot establish treatment efficacy or pregnancy outcome causality. They highlight the need for preconception counseling, individualized risk-benefit assessment, careful use of pregnancy-compatible maintenance therapy when clinically indicated, and multidisciplinary follow-up. Isolated weak MOG-IgA positivity requires cautious interpretation.

## 1 Introduction

Neuromyelitis optica spectrum disorder (NMOSD) is a rare, relapsing autoimmune disease of the central nervous system. It is defined by a group of core clinical syndromes, including optic neuritis, acute myelitis, area postrema syndrome, and brainstem or cerebral syndromes (Wingerchuk et al., 2015; Jarius et al., 2020). NMOSD attacks often involve the optic nerves and spinal cord and may result in severe, sometimes irreversible neurological disability (Wingerchuk et al., 2015). NMOSD predominantly affects women, many of whom are of reproductive age. Pregnancy planning is therefore an important component of long-term disease management (Jarius et al., 2020; Wang et al., 2022).

Pregnancy and the postpartum period require careful pharmacological decision-making in NMOSD. Relapse prevention is essential, as NMOSD attacks may cause substantial motor, sensory, visual, or autonomic disability, and pregnancy-related attacks have been documented in observational studies (Wang et al., 2020; Wang et al., 2022). However, concerns about fetal drug exposure may lead patients and clinicians to avoid or discontinue immunosuppressive therapy. In AQP4-IgG-positive NMOSD, this risk-benefit assessment is further complicated by evidence linking AQP4-IgG to placental injury and adverse pregnancy outcomes (Saadoun et al., 2013; Nour et al., 2016).

From a pharmacological perspective, the central issue is not whether immunosuppressive therapy should be stopped in all pregnant patients with NMOSD. Instead, treatment should be individualized according to disease activity, gestational age, fetal safety, maternal risk of disability, and patient values (Borisow et al., 2018; Leite et al., 2023). We report two contrasting pregnancies in women with NMOSD to illustrate decision-making in acute relapse treatment, maintenance immunosuppression, antibody-informed counseling, and postpartum surveillance.

## 2 Case presentation

### 2.1 Case 1: Severe relapse during early pregnancy followed by pregnancy termination

A 30-year-old primigravida was admitted at 18+3 weeks of gestation for planned pregnancy termination. Eight years earlier, she had developed bilateral lower-limb weakness, gait difficulty, sensory disturbance, nausea, vomiting, visual symptoms, and bowel and urinary dysfunction. An inflammatory demyelinating disorder with spinal cord and nerve root involvement was considered. Guillain-Barré syndrome had initially been included in the differential diagnosis. Her symptoms improved after high-dose corticosteroid therapy followed by oral corticosteroids and rehabilitation. During the following years, she experienced several relapsing neurological episodes, including dizziness, gait instability, visual blurring, lower-limb sensory disturbance, neuropathic pain, and cognitive symptoms. These episodes improved partially after corticosteroid-based treatment, but residual gait disturbance and distal lower-limb sensory symptoms persisted.

The current pregnancy was conceived spontaneously. Early prenatal assessment showed a normal nuchal translucency measurement, and no invasive prenatal diagnostic procedure had been performed. During the first trimester, she developed severe hyperemesis gravidarum with poor oral intake. At 11 weeks of gestation, she developed progressive bilateral lower-limb pain and weakness. The symptoms persisted for more than 10 days and led to inability to walk independently. She was admitted to the Department of Neurology for urgent evaluation and treatment.

Neurological examination showed preserved consciousness and normal cranial nerve findings. Muscle strength was Medical Research Council grade 5 in both upper limbs, grade 4 proximally and grade 2 distally in both lower limbs. Lower-limb muscle tone was increased. Bilateral pes cavus and impaired ankle dorsiflexion were noted. Hyperalgesia was present over the lower back, lower legs, and dorsum of the feet. Lower-limb tendon reflexes were reduced, and bilateral pathological signs were positive. Bladder ultrasonography showed post-void residual urine.

Brain and spinal magnetic resonance imaging showed a focal long-segment abnormal signal in the thoracic spinal cord. Mild cervical cord atrophy at the C4–C6 levels, with equivocal high signal, was also suspected. Brain MRI showed mild white matter hyperintensities and possible mild bilateral frontal lobe atrophy. Visual evoked potentials showed delayed and low-amplitude bilateral P100 responses. Electromyography showed multifocal peripheral nerve involvement in both lower limbs, affecting sensory and motor fibers, with progression compared with a previous electrophysiological study.

Lumbar puncture showed an opening pressure of 100 mmH_2_O. Routine cerebrospinal fluid testing, biochemical analysis, smear, culture, and IgG synthesis rate were unremarkable. Cerebrospinal fluid next-generation sequencing and oligoclonal bands were negative. Testing for central nervous system demyelinating antibodies showed negative serum AQP4-IgG and MOG-IgG, but weak serum MOG-IgA positivity at a titer of 1:10. Cerebrospinal fluid antibody testing was negative. The available records did not indicate whether repeat testing was performed or whether a live cell-based assay or enzyme-linked immunosorbent assay was used.

The working diagnosis was AQP4-IgG-negative NMOSD with weak isolated MOG-IgA positivity of uncertain clinical significance, rather than definite MOG antibody-associated disease. The diagnosis was supported by a relapsing course, acute myelitis with urinary dysfunction, a long-segment thoracic cord lesion, previous visual symptoms with abnormal visual evoked potentials, negative cerebrospinal fluid infection studies, and the absence of a better alternative diagnosis. Multiple sclerosis was considered less likely because typical multiple sclerosis-like brain MRI findings and oligoclonal bands were absent. Guillain-Barré syndrome or chronic inflammatory demyelinating polyneuropathy had been considered earlier because of lower-limb weakness and peripheral nerve involvement, but the spinal cord lesion, pathological signs, urinary dysfunction, and steroid-responsive relapsing course favored a central inflammatory demyelinating disorder.

After discussion with the patient and her family, she received intravenous methylprednisolone 1,000 mg/day for 5 days, followed by an oral corticosteroid taper. At the time of obstetric admission, she was taking prednisone acetate 50 mg/day. Formal Expanded Disability Status Scale (EDSS) and modified Rankin Scale scores were not prospectively recorded. Based on the available records, disability at relapse nadir was retrospectively estimated as mRS score 4 and approximate EDSS category 6.0–6.5, because she was unable to walk independently and had urinary dysfunction. After treatment, recovery was partial, with residual gait and sensory symptoms. These estimates should be interpreted with caution.

Given residual disability, concerns about fetal drug exposure, possible need for further immunosuppression, and anticipated rehabilitation burden during pregnancy, she was referred for multidisciplinary counseling. The counseling involved neurology, obstetrics, clinical pharmacy, and genetic counseling teams. At 16+2 weeks of gestation, she received counseling regarding the potential risks of medication exposure and the uncertainty surrounding pregnancy continuation. After further consideration, she and her family chose pregnancy termination. Labor induction was performed at 18+3 weeks of gestation. She subsequently continued neurological follow-up for long-term disease control.

### 2.2 Case 2: Stable pregnancy during continued prednisolone and azathioprine therapy

A 35-year-old primigravida presented to our hospital for prenatal care at 32 weeks of gestation. Six years earlier, she had developed acute visual impairment consistent with optic neuritis. Spinal magnetic resonance imaging revealed longitudinally extensive transverse myelitis across multiple spinal segments. Serum AQP4-IgG was positive, supporting a diagnosis of AQP4-IgG-positive NMOSD. She received high-dose corticosteroids and intravenous immunoglobulin, followed by clinical improvement. After 1 year of maintenance therapy, treatment was discontinued, and she remained relapse-free for a period.

Two years later, she experienced a relapse that was controlled after reinitiation of immunosuppressive therapy. After preconception counseling 1 year before conception, prednisolone 10 mg/day and azathioprine 50 mg/day were continued because this regimen had maintained clinical stability. Azathioprine 50 mg/day was interpreted as a low maintenance dose rather than a standard weight-based dose. Her pre-pregnancy body weight was not available in the referral records, so a reliable weight-adjusted dose could not be calculated. She conceived spontaneously while receiving this regimen.

**FIGURE 1:**
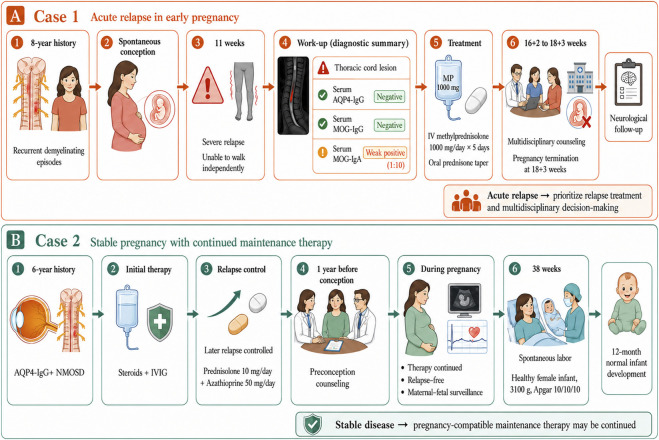
Clinical pharmacological decision pathways in two pregnancies complicated by NMOSD.

During pregnancy, monitoring included relapse symptoms, blood pressure, glucose levels, blood counts, liver function, infection, and fetal growth. She remained relapse-free, with no major obstetric complications and fetal growth appropriate for gestational age. At 38 weeks of gestation, she delivered a healthy female infant weighing 3,100 g, with Apgar scores of 10, 10, and 10 at 1, 5, and 10 min, respectively. Prednisolone and azathioprine were continued postpartum because early postpartum relapse risk was considered clinically relevant. At 12 months, the infant showed normal growth and age-appropriate development, with no reported congenital anomalies, severe infections, or neurological abnormalities. The clinical and pharmacological decision pathways are summarized in Table 1 and Figure 1.

**TABLE 1:** Key clinical and pharmacological contrasts between the two NMOSD pregnancies.

Characteristic	Case 1	Case 2
Maternal profile	30-year-old primigravida	35-year-old primigravida
Antibody status	AQP4-IgG negative; isolated weak MOG-IgA positivity at 1:10 of uncertain significance	AQP4-IgG positive
Diagnostic context	Relapsing course, acute myelitis, urinary dysfunction, long-segment thoracic cord lesion, abnormal visual evoked potentials	Established diagnosis before pregnancy; previous optic neuritis and longitudinally extensive transverse myelitis
Preconception status	No documented preconception stabilization or planned pregnancy-compatible maintenance therapy	Stable for 1 year on prednisolone and azathioprine
Pregnancy course	Severe relapse at 11 weeks with inability to walk independently and post-void residual urine	Relapse-free pregnancy under multidisciplinary surveillance
Disability assessment	Retrospective mRS score 4 at relapse nadir; approximate EDSS category 6.0–6.5	No relapse-related disability during pregnancy
Acute relapse treatment	Intravenous methylprednisolone 1,000 mg/day for 5 days, followed by oral corticosteroid tapering	Not required
Azathioprine dose context	Not applicable	Low maintenance dose; pre-pregnancy body weight unavailable, so weight-adjusted dose could not be calculated
Maintenance therapy during pregnancy	No planned maintenance regimen before relapse; oral prednisone acetate 50 mg/day after pulse therapy	Prednisolone 10 mg/day plus azathioprine 50 mg/day continued throughout pregnancy
Main counseling issue	Neurological disability, fetal drug exposure, rehabilitation burden, and disease uncertainty	Relapse prevention, fetal monitoring, and continuation of pregnancy-compatible therapy
Pregnancy outcome	Termination at 18+3 weeks after shared decision-making	Spontaneous delivery at 38 weeks
Infant outcome	Not applicable	Female infant weighing 3,100 g; apgar scores 10, 10 and 10; normal development at 12 months
Clinical-pharmacological message	Uncontrolled disease may lead to severe relapse and complex pregnancy counseling	Planned continuation of an effective regimen may support maternal stability, but safety cannot be inferred from a single case

Abbreviations: AQP4-IgG, aquaporin-4, immunoglobulin G; MOG-IgA, myelin oligodendrocyte glycoprotein immunoglobulin A; NMOSD, neuromyelitis optica spectrum disorder; EDSS, expanded disability status scale; mRS, modified Rankin Scale.Case 1 should not be classified as definite MOG antibody-associated disease based only on isolated weak MOG-IgA positivity. The table summarizes clinical decision pathways rather than causal effects of treatment.

## 3 Discussion

### 3.1 Case-based pharmacological message and preconception planning

These two cases should not be interpreted as matched or directly comparable cases. They represent two different clinical scenarios in NMOSD pregnancy. Case 1 involved delayed diagnostic clarification, no established long-term relapse-preventive therapy before pregnancy, severe relapse in early pregnancy, and diagnostic uncertainty related to weak isolated MOG-IgA positivity. This uncertainty is important because current NMOSD criteria require stronger clinical and MRI support in AQP4-IgG-negative patients, and current MOGAD criteria rely mainly on serum MOG-IgG tested by appropriate cell-based assays rather than isolated MOG-IgA positivity (Wingerchuk et al., 2015; Banwell et al., 2023). In contrast, Case 2 involved established AQP4-IgG-positive NMOSD, preconception disease stabilization, and continuation of a stable maintenance regimen.

Therefore, the different outcomes cannot be attributed to a single treatment decision. These cases cannot support causal conclusions about treatment efficacy or pregnancy outcome. Their value lies in showing how preconception disease control, relapse severity, diagnostic certainty, available treatment options, fetal safety concerns, and patient values may shape pharmacological decision-making during pregnancy. This framing is consistent with evidence that NMOSD attacks during pregnancy and the postpartum period may cause substantial disability, and that relapse-preventive treatment during pregnancy may reduce pregnancy-related attack risk in selected patients (Jarius et al., 2020; Wang et al., 2022; Leite et al., 2023).

From a clinical pharmacology perspective, these two scenarios raise four practical questions for NMOSD management during pregnancy. These questions concern preconception optimization of disease control, treatment of acute relapse during pregnancy, continuation of maintenance therapy considered acceptable during pregnancy, and postpartum relapse prevention. Preconception counseling is especially important. Agents with established teratogenic or fetotoxic potential, including mycophenolate mofetil, methotrexate, and cyclophosphamide, should be discontinued before conception and replaced with pregnancy-appropriate alternatives whenever clinically feasible (Russell et al., 2023; Kümpfel et al., 2024). In selected patients with clinically meaningful relapse risk, continuation of pregnancy-compatible relapse-preventive therapy may be preferable to unplanned treatment withdrawal after shared counseling (Wang et al., 2022; Leite et al., 2023; Kümpfel et al., 2024).

### 3.2 Treatment of acute relapse during pregnancy

Disabling relapse during pregnancy requires urgent clinical assessment and timely treatment. High-dose intravenous methylprednisolone is commonly used for acute NMOSD relapse and may be considered during pregnancy when the expected maternal benefit outweighs potential fetal risk (Borisow et al., 2018; D’Souza et al., 2020). In Case 1, high-dose methylprednisolone was chosen because the patient had disabling motor impairment, sensory symptoms, and urinary dysfunction, with MRI and electrophysiological findings supporting active neurological involvement. For severe corticosteroid-refractory attacks, intravenous immunoglobulin or plasma exchange may be considered after multidisciplinary evaluation, particularly when the risk of persistent maternal neurological disability is high (Borisow et al., 2018; Leite et al., 2023).

Counseling should not present relapse treatment as a simple trade-off between maternal health and fetal safety. Clinicians should explain that severe maternal relapse may also compromise pregnancy through immobility, infection, poor nutritional intake, psychological distress, and progressive functional decline. Available epidemiological data have not shown a clear increase in oral clefts or major congenital malformations after early-pregnancy corticosteroid exposure. However, prolonged corticosteroid therapy still requires careful maternal monitoring for hypertension, hyperglycemia, infection risk, and bone-related complications (Bay Bjørn et al., 2014; Committee on Obstetric Practice and Society for Maternal-Fetal Medicine, 2019). Treatment decisions should therefore consider gestational age, relapse severity, likelihood of neurological recovery, fetal drug exposure, maternal functional prognosis, available rescue options, and patient values.

Pregnancy termination in Case 1 should not be interpreted as a general recommendation for NMOSD relapse during pregnancy. It was an individualized decision made by the patient and her family after multidisciplinary counseling, within the local legal, ethical, and clinical context. Counseling was influenced by severe neurological impairment, residual disability after acute relapse treatment, uncertainty about further immunosuppressive therapy, concerns about fetal drug exposure, and the expected burden of prolonged rehabilitation during pregnancy.

International readers should note that decisions about pregnancy continuation or termination may differ across countries and communities because of legal frameworks, cultural values, religious views, health-system resources, and patient preferences. This is consistent with the broader principle that reproductive healthcare should be evidence-based, person-centered, and responsive to the values and circumstances of the pregnant individual (World Health Organization, 2022). In many settings, pregnancy continuation with aggressive relapse treatment, rescue therapy such as plasma exchange or intravenous immunoglobulin, rehabilitation support, and pregnancy-compatible maintenance immunosuppression may be the preferred or only acceptable pathway (Borisow et al., 2018; Leite et al., 2023; Kümpfel et al., 2024). Therefore, this case is best understood as an example of shared decision-making under severe clinical uncertainty, rather than as a model for universal practice.

### 3.3 Maintenance therapy and biologic options in pregnancy

Case 2 illustrates the practical value of maintenance therapy in selected pregnant patients with NMOSD, but this observation should be interpreted with caution. A single favorable pregnancy cannot establish the safety or efficacy of prednisolone plus azathioprine. In this patient, continuation of prednisolone 10 mg/day and azathioprine 50 mg/day was chosen because the regimen had maintained clinical stability for approximately 1 year before conception. The azathioprine dose should be interpreted as a low maintenance dose rather than a standard weight-based dose. The pre-pregnancy body weight of patient was not available in the referral records, so a reliable weight-adjusted dose could not be calculated.

Azathioprine is not specifically approved for NMOSD and should be regarded as an off-label relapse-preventive therapy in this setting. However, it has been widely used as a conventional immunosuppressive agent in NMOSD practice, particularly before the availability of approved biologic therapies. In pregnancy, azathioprine has a more acceptable reproductive safety profile than known teratogenic immunosuppressants such as mycophenolate mofetil, methotrexate, and cyclophosphamide (Borisow et al., 2018; Russell et al., 2023; Kümpfel et al., 2024). Its continuation may be considered when maternal relapse risk is clinically meaningful, but this decision requires shared counseling and maternal, fetal, and neonatal monitoring.

The treatment landscape for NMOSD has changed rapidly. Approved biologic options for AQP4-IgG-positive NMOSD now include complement C5 inhibitors, IL-6 receptor blockade, and CD19-directed therapy in many regions. CD20-directed B-cell depletion is also used in NMOSD practice in selected regions or as off-label therapy. These agents provide important alternatives to conventional immunosuppression, but their use in pregnancy requires careful interpretation (Leite et al., 2023; Kümpfel et al., 2024). Pregnancy experience with C5 inhibitors is relatively more reassuring than that with several other biologic agents, mainly from paroxysmal nocturnal hemoglobinuria, atypical hemolytic uremic syndrome, and limited NMOSD reports. Pregnancy data for satralizumab remain limited, although case reports and pregnancy registries are emerging. For CD19^−^and CD20-directed therapies, fetal and neonatal B-cell depletion, infection risk, blood-count monitoring, and live-vaccine timing are important concerns.

Therefore, biologic therapies should not be discussed as uniformly safe or unsafe during pregnancy. Their use should be individualized according to maternal relapse risk, antibody status, prior treatment response, gestational age, placental transfer, local drug availability, patient preference, and neonatal monitoring capacity. *Postartum* management should also be planned before delivery, because relapse risk may increase during the early postpartum period. Maintenance therapy, breastfeeding plans, infection surveillance, neurological follow-up, infant blood-count monitoring when indicated, and vaccination planning should be addressed through multidisciplinary counseling (Wang et al., 2022; Leite et al., 2023; Kümpfel et al., 2024).

### 3.4 Antibody status, diagnostic uncertainty, and pregnancy counseling

Antibody status is a key component of counseling and risk assessment in pregnant patients with NMOSD. AQP4-IgG-positive NMOSD has important implications for pregnancy. AQP4 is expressed in placental tissue, and experimental and clinical data suggest that AQP4-IgG may be associated with placental inflammation, fetal injury, miscarriage, and other adverse pregnancy outcomes (Saadoun et al., 2013; Nour et al., 2016; Jarius et al., 2020; Leite et al., 2023).

By contrast, Case 1 underscores the diagnostic uncertainty created by a weak serum MOG antibody result. Available clinical records showed a low-titer serum MOG antibody result of 1:10. Cerebrospinal fluid testing for demyelination-associated antibodies was negative. However, the MOG antibody isotype, assay platform, and whether repeat testing had been performed were not clearly documented. This distinction is clinically important because current diagnostic frameworks for NMOSD and MOG antibody-associated disease rely mainly on AQP4-IgG and MOG-IgG status, especially when these antibodies are tested with appropriate cell-based assays. In contrast, isolated or low-titer MOG antibody findings with an uncertain isotype should be interpreted with caution (Wingerchuk et al., 2015; Jarius et al., 2020; Banwell et al., 2023; Leite et al., 2023). Therefore, this case should not be classified as definite MOG antibody-associated disease. It is better described as AQP4-IgG-negative NMOSD with a weak serum MOG antibody result of uncertain clinical significance.

### 3.5 Strengths, limitations, and clinical implications

This report presents two real-world clinical scenarios at the intersection of neurology, obstetrics, and clinical pharmacology. It highlights how maternal relapse risk, fetal drug exposure, diagnostic certainty, patient preference, and local clinical context may shape NMOSD treatment decisions during pregnancy.

Several limitations should be acknowledged. The two cases were not directly comparable, and no causal conclusion can be drawn. Case 1 involved diagnostic uncertainty because isolated weak MOG-IgA positivity has unclear significance. Antibody assay details, repeat testing, and formal prospective disability scores were unavailable. In Case 2, pre-pregnancy body weight was unavailable, so a reliable weight-adjusted azathioprine dose could not be calculated. Infant follow-up was limited to 12 months. These limitations support the need for prospective NMOSD pregnancy registries.

These limitations highlight the need for prospective pregnancy registries in NMOSD. Future studies should record antibody testing methods, standardized disability scores, treatment timing, dose adjustment, maternal relapse outcomes, breastfeeding exposure, infant immune monitoring, vaccination planning, and long-term child development.

## 4 Conclusion

These two clinical scenarios highlight the pharmacological complexity of NMOSD management during pregnancy. They are not directly comparable, and no causal conclusion could be drawn regarding treatment efficacy or pregnancy outcome. Case 1 illustrates difficult counseling after severe early-pregnancy relapse in the setting of diagnostic uncertainty and no established relapse-preventive therapy. Case 2 illustrates relapse-free pregnancy in a clinically stable AQP4-IgG-positive patient who continued a stable maintenance regimen. Overall, these cases support preconception counseling, individualized risk-benefit assessment, careful use of pregnancy-compatible maintenance therapy when clinically indicated, and multidisciplinary follow-up. Weak isolated MOG-IgA positivity remains clinically uncertain.

## Data Availability

The raw data supporting the conclusions of this article will be made available by the authors, without undue reservation.
